# Regeneration of Biomechanically Functional Tendon Tissue Following Injection of Uncultured, Autologous, Adipose-Derived Regenerative Cells into Partial Achilles Tendon Defects in Rabbits

**DOI:** 10.3390/ijms26146800

**Published:** 2025-07-16

**Authors:** Christoph Schmitz, Christopher Alt, Tobias Wuerfel, Stefan Milz, Jacqueline Dinzey, Ashley Hill, Katie J. Sikes, Lindsey H. Burton, Jeremiah Easley, Holly L. Stewart, Christian M. Puttlitz, Benjamin C. Gadomski, Kevin M. Labus, David A. Pearce, Nicola Maffulli, Eckhard U. Alt

**Affiliations:** 1 Institute of Anatomy, Faculty of Medicine, LMU Munich, 80331 Munich, Germany; christopher.alt@isarklinikum.de (C.A.); tobias.wuerfel@mri.tum.de (T.W.); stefan.milz@med.uni-muenchen.de (S.M.); 2 IsarKlinikum, 80331 Munich, Germany; ealt@tulane.edu; 3 InGeneron Inc., Houston, TX 77054, USA; jdinzey@ingeneron.com (J.D.); ashill@athenahealth.com (A.H.); 4 Department of Sports Orthopaedics, Technical University of Munich, 81677 Munich, Germany; 5 Preclinical Surgical Research Laboratory, Department of Clinical Sciences, C. Wayne McIlwraith Translational Medicine Institute, Colorado State University, Fort Collins, CO 80523, USA; katie.sikes@colostate.edu (K.J.S.); jeremiah.easley@colostate.edu (J.E.); holly.stewart@colostate.edu (H.L.S.); 6 Orthopaedic Bioengineering Research Laboratory, Department of Mechanical Engineering, Colorado State University, Fort Collins, CO 80524, USA; christian.puttlitz@colostate.edu (C.M.P.); ben.gadomski@colostate.edu (B.C.G.); kevin.labus@colostate.edu (K.M.L.); 7 Department of Pediatrics, Sanford School of Medicine, University of South Dakota, Sioux Falls, SD 57105, USA; david.pearce@usd.edu; 8 Department of Trauma and Orthopaedic Surgery, Faculty of Medicine and Psychology, University La Sapienza, 00185 Roma, Italy; n.maffulli@qmul.ac.uk; 9 School of Pharmacy and Bioengineering, Faculty of Medicine, Keele University, Stoke on Trent ST4 7QB, UK; 10 Centre for Sports and Exercise Medicine, Barts and the London School of Medicine and Dentistry, Mile End Hospital, Queen Mary University of London, London E1 4DG, UK; 11 Heart and Vascular Institute, Department of Medicine, Tulane University Health Science Center, New Orleans, LA 70112, USA

**Keywords:** animal model, partial tendon tears, tissue regeneration, UA-ADRCS, stem cells, stromal vascular fraction

## Abstract

Current treatment strategies for partial tendon tears often lack the capacity to promote true tissue regeneration and improve long-term clinical outcomes. This study tested the hypothesis that treatment of a partial defect in the rabbit common calcaneus tendon (CCT) with uncultured, unmodified, autologous, adipose-derived regenerative cells (UA-ADRCs) enables regenerative healing without scar formation. A full-thickness, 3 mm defect was produced in the midsubstance of the right gastrocnemius tendon, a component of the CCT, in adult female New Zealand white rabbits. Animals received either an injection of 28.3 × 10^6^ UA-ADRCs in 0.5 mL Ringer’s lactated solution (RLS) or saline, or RLS or saline alone as sham treatment. Tendons were analyzed 4 or 12 weeks post-treatment using histology, immunohistochemistry and non-destructive biomechanical testing. UA-ADRC-treated tendons showed newly formed connective tissue consistent with tendon regeneration, whereas sham-treated tendons developed scar tissue. Biomechanical testing showed significantly higher percent relaxation in UA-ADRC-treated tendons compared to sham controls (*p* < 0.05), indicating greater viscoelasticity characteristic of healthy or well-integrated tissue. Together, these findings suggest that UA-ADRC therapy may provide a regenerative, structure-modifying treatment for partial tendon tears.

## 1. Introduction

Current clinical treatment options for tendon tears offer only limited potential for true tissue regeneration and improvement in clinical outcomes. Treatment of symptomatic, partial-thickness rotator cuff tears (sPTRCTs) with uncultured, unmodified, autologous, adipose-derived regenerative cells (UA-ADRCs), isolated from lipoaspirate at the point of care, is safe and more effective than corticosteroid injection [1,2]. In a randomized clinical trial (RCT), subjects aged 30 to 75 years with sPTRCT who did not respond to at least six weeks of physical therapy received either a single injection of an average 11.4 × 10^6^ UA-ADRCs (in 5 mL solution; mean cell viability: 88%) or a single injection of 80 mg methylprednisolone (40 mg/mL; 2 mL) combined with 3 mL of 0.25% bupivacaine [1,2]. UA-ADRCs were enzymatically isolated from autologous lipoaspirate at the point of care using the Transpose RT system (InGeneron Inc., Houston, TX, USA) [3,4,5]. No serious adverse events related to UA-ADRC injection occurred during the follow-up. The risk profile of UA-ADRC treatment was no greater than that of corticosteroid injection. Compared to corticosteroids, UA-ADRC treatment led to significantly higher ASES Total scores at weeks 24 and 52 and month 41, significantly higher SF-36 Total scores at week 24, and significantly improved VAS Pain scores at weeks 24 and 52 (*p* < 0.05) [1,2]. Thus, UA-ADRC therapy for sPTRCT appears safe, and improves shoulder function without adverse effects. To confirm these findings in a larger population, an RCT with 168 sPTRCT patients is currently underway [6].

In animal models, injection of adult stem cells derived from adipose tissue into injured tendons has shown promising biological effects [7,8,9,10], including reduced inflammation, enhanced tendon regeneration with minimal scarring, better collagen fiber organization, and improved mechanical properties such as greater load-to-failure and tensile strength. However, it remained unclear whether similar regenerative effects could be demonstrated in human tendon tissue treated with UA-ADRCs. To address this, we recently conducted the first comprehensive histological and immunohistochemical analysis of a supraspinatus tendon biopsy taken from a 66-year-old patient ten weeks after local injection of UA-ADRCs for a traumatic rotator cuff injury [11]. The UA-ADRCs were isolated at the point of care using the Transpose RT system (InGeneron) [1,2,3,4,5,6]. The analysis revealed clear signs of regenerative healing of the injured tendon, with no evidence of adipocyte formation, indicating that UA-ADRCs can generate new tendon tissue and contribute to tendon regeneration in humans.

Building on these findings, the present study tested, for the first time, the hypothesis that treatment of a partial defect in the rabbit common calcaneal tendon (CCT) with UA-ADRCs isolated from rabbit adipose tissue using the Transpose RT system (InGeneron) promotes faster and more effective tendon regeneration compared to treatment with Ringer’s lactated solution (RLS). The defect was created by punching a 3 mm full-thickness hole in the midsubstance of the right gastrocnemius tendon, a component of the CCT. Rabbits were chosen as they provide a suitable intermediate model for studying tendon regeneration, offering a balance between small rodents and large mammals [12]. The structural characteristics of their tendons closely approximate those of human tendons, making them valuable for investigating surgical techniques, tendon healing processes and pathologies such as tendinopathy [12,13]. Furthermore, similar to UA-ADRCs enzymatically isolated from human adipose tissue [3], ADRCs derived from rabbit adipose tissue also exhibit trilineage differentiation potential, demonstrating the ability to differentiate into somatic cells of all three germ layers [14]. Moreover, the surface marker profile of rabbit-derived ADRCs (specifically CD14, CD31, CD34, CD45, CD90 and CD146) [15,16,17,18,19] resembles that of UA-ADRCs isolated from human lipoaspirate using the Transpose RT system (InGeneron) [5].

Based on these similarities, we further hypothesized that the histological and immunohistochemical findings in this animal model would mirror those previously observed in the human supraspinatus tendon [11], and that the biomechanical properties of the CCT would be better restored following UA-ADRC treatment than after sham treatment.

## 2. Results

### 2.1. Histology

**Figure 1:**
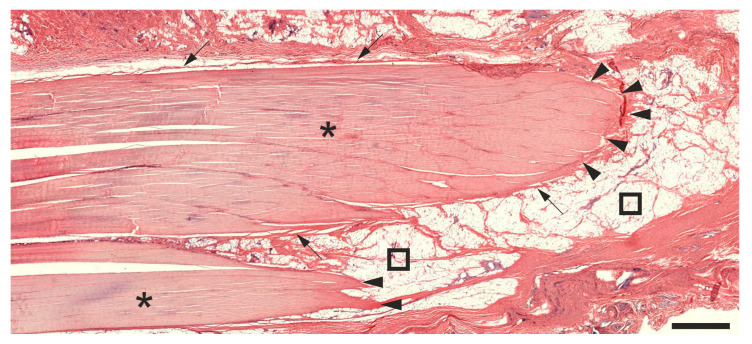
Low-power photomicrograph of a representative 5 µm thick, H&E-stained section of the left common calcaneal tendon from a rabbit without surgery or treatment. Further details are provided in the text. The scale bar represents 1 mm. Asterisks indicate tendon tissue, arrows point to the peritenon, and squares mark surrounding adipose tissue. Arrowheads highlight regions where the CCT left the plane of section.

For comparison, Figure 1 presents a representative photomicrograph of a 5 µm thick, H&E-stained section from a left CCT of a rabbit in Group 1 (no surgery, no treatment).

**Figure 2:**
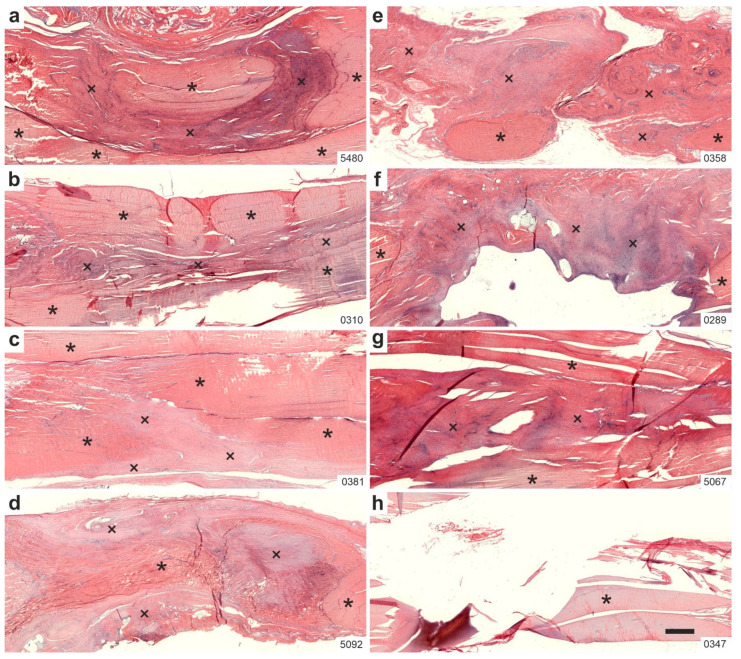
Representative low-power photomicrographs of 5 µm thick, H&E-stained sections of the right common calcaneal tendon (surgery, treatment or sham treatment side) from rabbits in Group 1 (UA-ADRCs/W4; (**a**–**d**)) and Group 3 (sham/W4; (**e**–**h**)). Asterisks indicate original tendon tissue; crosses denote newly formed connective tissue. Numbers in the lower right corner of each panel indicate individual animal IDs according to the study protocol. The scale bar in panel (**h**) represents 1 mm and applies to all panels. Further details are provided in the text.

Figure 2 shows representative low-power photomicrographs of 5 µm thick, H&E-stained sections from the right CCT of rabbits in Group 1 (treatment with UA-ADRCs, four weeks post-treatment; hereafter referred to as UA-ADRCs/W4) (Figure 2a–d), and in Group 3 (sham treatment, four weeks post-treatment; sham/W4) (Figure 2e–h).

At low magnification, Group 1 (UA-ADRCs/W4) sections revealed newly formed connective tissue that completely filled the gap between the original tendon stumps and was homogeneously integrated into the tendon’s longitudinal structure (Figure 2a–d). In contrast, Group 3 (sham/W4) sections showed newly formed connective tissue that neither completely bridged the gap between tendon stumps nor integrated homogeneously into the tendon structure (Figure 2e–h).

**Figure 3:**
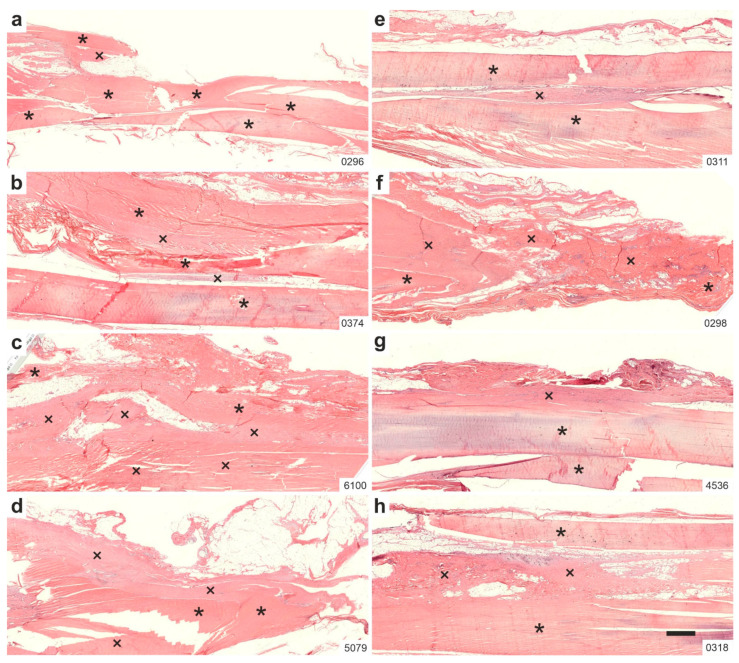
Representative low-power photomicrographs of 5 µm thick, H&E-stained sections of the right common calcaneal tendon (surgery, treatment or sham treatment side) from rabbits in Group 2 (UA-ADRCs/W12) (**a**–**d**) and Group 4 (sham/W12) (**e**–**h**). Asterisks indicate original tendon tissue; crosses denote newly formed connective tissue. Numbers in the lower right corner of each panel indicate individual animal IDs according to the study protocol. The scale bar in panel (**h**) represents 1 mm and applies to all panels. Further details are provided in the text.

Figure 3 displays representative low-power photomicrographs of 5 µm thick, H&E-stained sections from the right CCT of rabbits in Group 2 (UA-ADRCs, 12 weeks post-treatment; UA-ADRCs/W12) (Figure 3a–d), and in Group 4 (sham treatment, 12 weeks post-treatment; sham/W12) (Figure 3e–h).

In Group 2 (UA-ADRCs/W12) sections, it was difficult to distinguish between the original tendon tissue and the newly formed connective tissue, as both were in close contact (Figure 3a–d). In contrast, Group 4 (sham/W12) sections allowed for a much clearer distinction between the original tendon and the newly formed tissue (Figure 3e–h).

**Figure 4:**
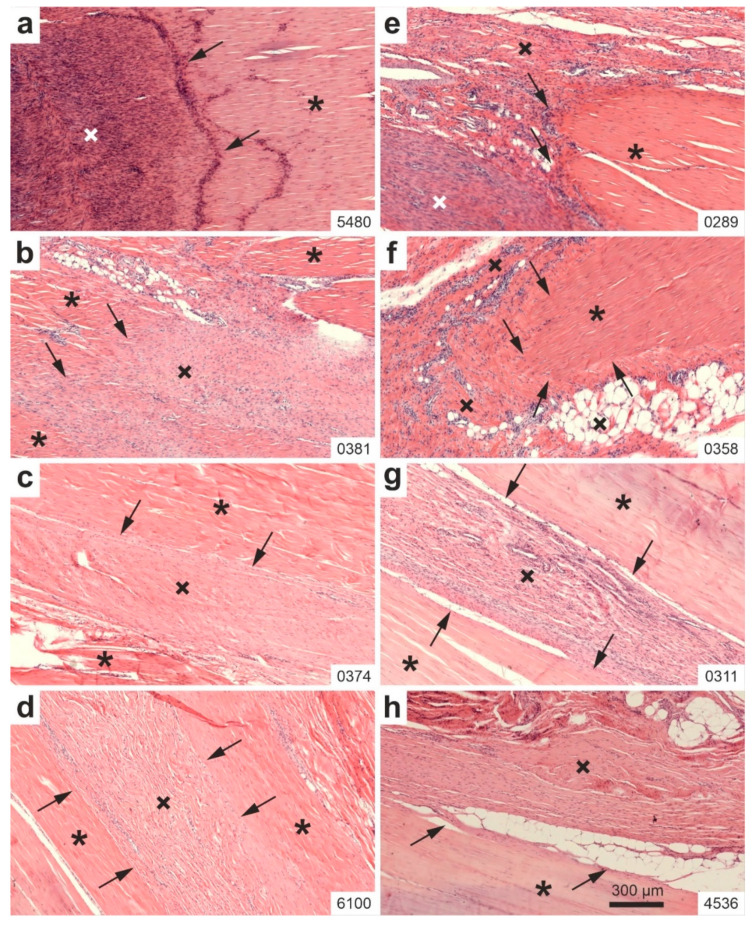
Representative high-power photomicrographs of 5 µm thick, H&E-stained sections of the right common calcaneal tendon (surgery, treatment or sham treatment side) from two rabbits each in Group 1 (UA-ADRCs/W4) (**a**,**b**), Group 2 (UA-ADRCs/W12) (**c**,**d**), Group 3 (sham/W4) (**e**,**f**), and Group 4 (sham/W12) (**g**,**h**). Asterisks indicate original tendon tissue; black and white crosses denote newly formed connective tissue; arrows mark the boundary between the original tendon and the newly formed tissue. Numbers in the lower right corner of each panel indicate individual animal IDs according to the study protocol. The scale bar in panel (**h**) represents 300 µm and applies to all panels. Further details are provided in the text.

Figure 4 presents representative high-power photomicrographs of 5 µm thick, H&E-stained sections from the right CCT of two rabbits per group: Group 1 (UA-ADRCs/W4) (Figure 4a,b), Group 2 (UA-ADRCs/W12) (Figure 4c,d), Group 3 (sham/W4) (Figure 4e,f), and Group 4 (sham/W12) (Figure 4g,h).

At higher magnification, Group 1 (UA-ADRCs/W4) and Group 2 (UA-ADRCs/W12) showed close contact between the original tendon and the newly formed connective tissue, without any evidence of blood vessel formation at the junction (Figure 4a–d). In contrast, Group 3 (sham/W4) showed vascularization at the interface (Figure 4e,f), and Group 4 (sham/W12) displayed adipose tissue formation between the tendon and the newly formed tissue, indicating a lack of close tissue contact (Figure 4g,h).

Furthermore, in rabbits treated with UA-ADRCs (Groups 1 and 2), the orientation of cells and extracellular matrix (ECM) within the newly formed connective tissue mirrored that of the original tendon. This organized alignment was absent in Groups 3 and 4 (sham), where the newly formed tissue lacked any clear orientation of cells and ECM after sham treatment.

### 2.2. Polarization Microscopy

**Figure 5:**
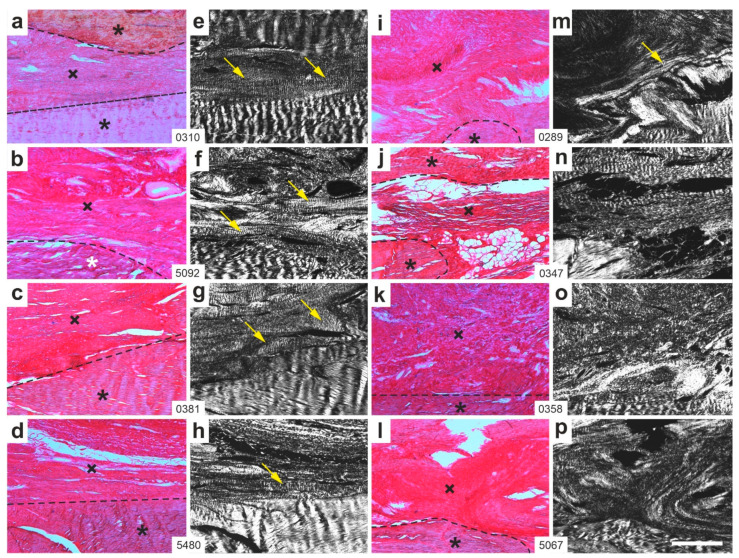
Representative polarization photomicrographs of 5 µm thick sections of the right common calcaneal tendon (surgery, treatment or sham treatment side) from rabbits in Group 1 (UA-ADRCs/W4) (**a**–**h**) and Group 3 (sham/W4) (**i**–**p**), stained with Picrosirius Red. Panels (**a**–**d**,**i**–**l**) were captured under brightfield microscopy; panels (**e**–**h**,**m**–**p**) show the corresponding fields of view imaged under polarization microscopy. Black and white asterisks mark original tendon tissue; crosses indicate newly formed connective tissue; dashed lines delineate the boundary between the two. Yellow arrows highlight areas of organized, firm connective tissue with visible crimp patterns within the newly formed tissue. Numbers in the white boxes linking each pair of corresponding panels (**a**/**e**,**b**/**f**,**c**/**g** … **l**/**p**) indicate individual animal IDs according to the study protocol. The scale bar in panel (**p**) represents 500 µm and applies to all panels. Further details are provided in the text.

Figure 5 presents representative polarization photomicrographs of 5 µm thick sections stained with Picrosirius Red from the right CCT of rabbits in Group 1 (UA-ADRCs/W4) (Figure 5a–h) and Group 3 (sham/W4) (Figure 5i–p).

At four weeks post-treatment, all rabbits in Group 1 (UA-ADRCs/W4) exhibited newly formed, organized and firm connective tissue with a discernible crimp pattern (Figure 5e–h). In contrast, only one rabbit in Group 3 (sham/W4; Figure 5m) showed a comparable degree of organized connective tissue formation.

**Figure 6:**
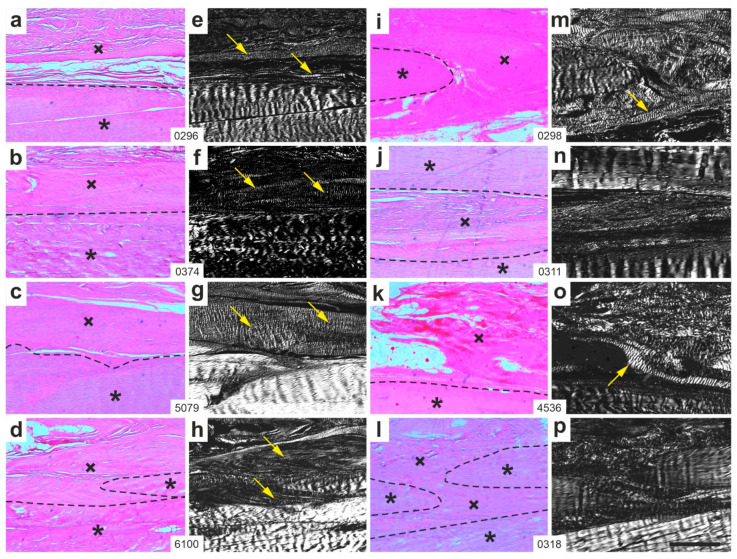
Representative polarization photomicrographs of 5 µm thick sections of the right common calcaneal tendon (surgery, treatment or sham treatment side) from rabbits in Group 2 (UA-ADRCs/W12) (**a**–**h**) and Group 4 (sham/W12) (**i**–**p**), stained with Picrosirius Red. Panels (**a**–**d**,**i**–**l**) were captured under brightfield microscopy; panels (**e**–**h**,**m**–**p**) show the corresponding fields of view imaged under polarization microscopy. Asterisks mark original tendon tissue; crosses indicate newly formed connective tissue; dashed lines delineate the boundary between the two. Yellow arrows highlight areas of organized, firm connective tissue with visible crimp patterns within the newly formed tissue. Numbers in the white boxes linking each pair of corresponding panels (**a**/**e**,**b**/**f**,**c**/**g** … **l**/**p**) indicate individual animal IDs according to the study protocol. The scale bar in panel (**p**) represents 500 µm and applies to all panels. Further details are provided in the text.

Figure 6 shows representative polarization photomicrographs of 5 µm thick sections stained with Picrosirius Red from the right CCT of rabbits in Group 2 (UA-ADRCs/W12) (Figure 6a–h) and Group 4 (sham/W12) (Figure 6i–p).

At 12 weeks post-treatment, all rabbits in Group 2 (ADRCs/W12) displayed newly formed, organized and firm connective tissue with visible crimp structure (Figure 6e–h). In Group 4 (sham/W12); however, only two rabbits (Figure 6m,o) demonstrated such tissue organization.

### 2.3. Immunolabeling of Type I Procollagen

**Figure 7:**
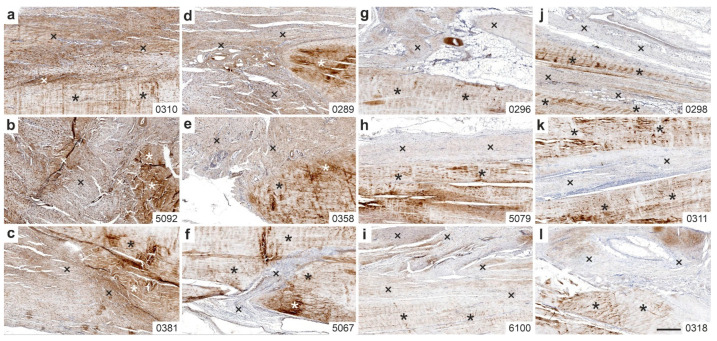
Representative photomicrographs of immunohistochemical detection of type I procollagen in 5 µm thick sections of the right common calcaneal tendon (surgery, treatment or sham treatment side) from three rabbits each in Group 1 (UA-ADRCs/W4) (**a**–**c**), Group 3 (sham/W4) (**d**–**f**), Group 2 (UA-ADRCs/W12) (**g**–**i**) and Group 4 (sham/W12) (**j**–**l**). Black and white asterisks indicate original tendon tissue; crosses mark newly formed connective tissue. Numbers in the lower right corner of each panel indicate individual animal IDs according to the study protocol. The scale bar in panel (**l**) represents 500 µm and applies to all panels. Further details are provided in the text.

Figure 7 displays representative photomicrographs showing immunohistochemical detection of type I procollagen in 5 µm thick sections from the right CCT of three rabbits each in Group 1 (UA-ADRCs/W4) (Figure 7a–c), Group 3 (sham/W4) (Figure 7d–f), Group 2 (UA-ADRCs/W12) (Figure 7g–i), and Group 4 (sham/W12) (Figure 7j–l). The sections of the rabbits 5480 (UA-ADRCs/W4), 0347 (sham/W4), 0374 (UA-ADRCs/W12) and 4536 (sham/W12) did not contain either original tendon tissue or newly formed connective tissue and were therefore excluded from evaluation.

All examined rabbits in Group 1 (UA-ADRCs/W4) exhibited strong extracellular immunolabeling for type I procollagen within the newly formed connective tissue (Figure 7a–c). In Group 3 (sham/W4), two of the three rabbits also showed extracellular labeling, though markedly less intense than in Group 1 (Figure 7d,e). The third rabbit in Group 3 demonstrated almost no immunolabeling for type I procollagen in the newly formed tissue (Figure 7f).

All examined rabbits in Group 2 (UA-ADRCs/W12) showed extracellular immunolabeling for type I procollagen in the newly formed connective tissue (Figure 7g–i), although the signal was less pronounced compared to Group 1 (UA-ADRCs/W4). In contrast, all animals in Group 4 (sham/W12) exhibited minimal to no immunolabeling for type I procollagen in the newly formed connective tissue (Figure 7j–l).

### 2.4. Immunolabeling of Type III Collagen

**Figure 8:**
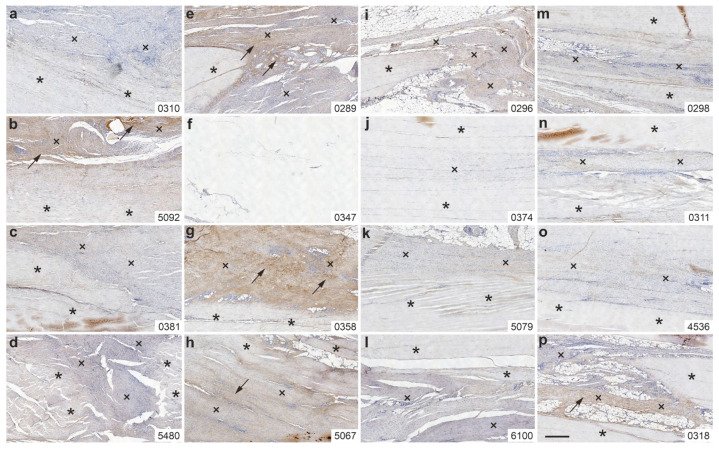
Representative photomicrographs of immunohistochemical detection of type III collagen in 5 µm thick sections of the right common calcaneal tendon (surgery, treatment or sham treatment side) from rabbits in Group 1 (UA-ADRCs/W4) (**a**–**d**), Group 3 (sham/W4) (**e**–**h**), Group 2 (UA-ADRCs/W12) (**i**–**l**), and Group 4 (sham/W12) (**m**–**p**). Asterisks denote original tendon tissue; crosses indicate newly formed connective tissue; arrows highlight immunolabeling for type III collagen within the newly formed tissue. Numbers in the lower right corner of each panel indicate individual animal IDs according to the study protocol. The scale bar in panel (**p**) represents 500 µm and applies to all panels. Further details are provided in the text.

Figure 8 shows representative photomicrographs of immunohistochemical detection of type III collagen in 5 µm thick sections from the right CCT of rabbits in Group 1 (UA-ADRCs/W4) (Figure 8a–d), Group 3 (sham/W4) (Figure 8e–h), Group 2 (UA-ADRCs/W12) (Figure 8i–l) and Group 4 (sham/W12) (Figure 8m–p).

Among the animals in Group 1 (UA-ADRCs/W4), one rabbit (Figure 8b) showed immunolabeling for type III collagen in the newly formed connective tissue. In Group 3 (sham/W4), three rabbits (Figure 8e,g,h) exhibited intense immunolabeling for type III collagen. The section from rabbit 0347 (Group 3), however, did not contain either original tendon tissue or newly formed connective tissue, and was therefore excluded from evaluation (Figure 8f).

In Group 4 (sham/W12), one rabbit showed discrete immunolabeling for type III collagen in the newly formed connective tissue (Figure 8p), while the remaining animals in this group, as well as all rabbits in Group 2 (UA-ADRCs/W12), showed no detectable immunolabeling for type III collagen.

### 2.5. Immunolabeling of CD163

**Figure 9:**
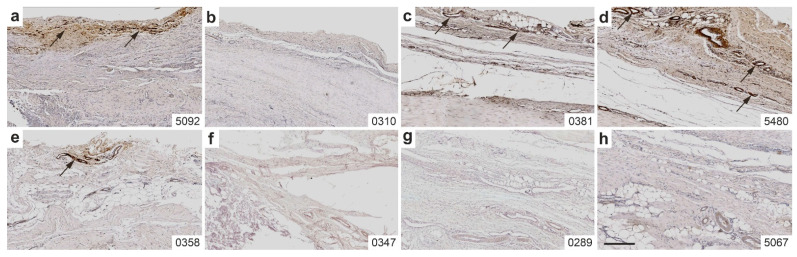
Representative photomicrographs of immunohistochemical detection of CD163 in 5 µm thick sections of the right common calcaneal tendon (surgery, treatment or sham treatment side) from rabbits in Group 1 (UA-ADRCs/W4) (**a**–**d**) and Group 3 (sham/W4) (**e**–**h**). Arrows indicate CD163-positive cells in the paratenon, predominantly located in proximity to blood vessels. Numbers in the lower right corner of each panel indicate individual animal IDs according to the study protocol. The scale bar in panel (**h**) represents 300 µm and applies to panels. Further details are provided in the text.

Figure 9 presents representative photomicrographs of immunohistochemical detection of CD163 in 5 µm thick sections of the right CCT from rabbits in Group 1 (UA-ADRCs/W4) (Figure 9a–d) and Group 3 (sham/W4) (Figure 9e–h).

CD163 immunolabeling was observed in the paratenon of the CCT in three animals from Group 1 (Figure 9a,c,d), and to a lesser extent in one animal from Group 3 (Figure 9e). The labeling was most prominently associated with blood vessels.

No CD163 immunolabeling was detected in the paratenon of the right CCT in animals from Group 2 (UA-ADRCs/W12) and Group 4 (sham/W12).

### 2.6. Immunolabeling of Aggrecan

**Figure 10:**
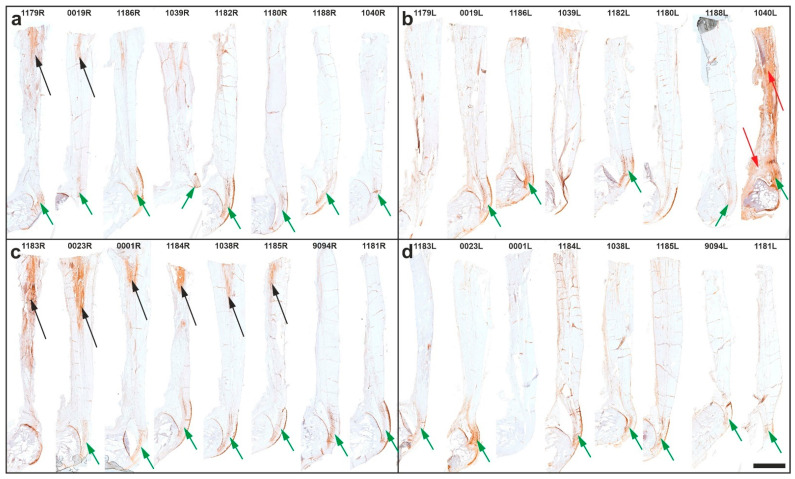
Representative photomicrographs of immunohistochemical detection of aggrecan in 5 µm thick sections of the common calcaneal tendon (CCT) from rabbits in Group 5 (UA-ADRCs/W12) (**a**,**b**) and Group 6 (sham/W12) (**c**,**d**). Panels a and c show the right CCT (surgery, treatment or sham treatment side); panels (**b**,**d**) show the left CCT (no surgery, no treatment or sham treatment). Green arrows indicate aggrecan detection at the distal part of the CCT; black arrows mark aggrecan at the surgical or treatment site; red arrows indicate a false-positive immunoreaction in the left CCT and paratenon tissue of rabbit 1040. Animal IDs according to the study protocol are noted above each photomicrograph. The scale bar in panel (**d**) represents 5 mm and applies to all panels. Further details are provided in the text.

Figure 10 shows overview photomicrographs of immunohistochemical detection of aggrecan in 5 µm thick sections of the right and left CCTs from all rabbits in Group 5 (UA-ADRCs/W12) (Figure 10a,b) and Group 6 (sham/W12) (Figure 10c,d).

In nearly all sections, immunolabeling for aggrecan was observed at the distal portion of the CCT, where the tendon is redirected by the posterosuperior corner of the calcaneus acting as a fulcrum—an area exposed to compressive forces (indicated by green arrows in Figure 10). Additionally, aggrecan immunolabeling was unexpectedly observed at the putative site of surgery and treatment or sham treatment in sections from six rabbits in Group 6 (sham/W12), but only two rabbits in Group 5 (UA-ADRCs/W12) (black arrows in Figure 10).

### 2.7. Combination of Polarization Microscopy and Immunohistochemistry

**Figure 11:**
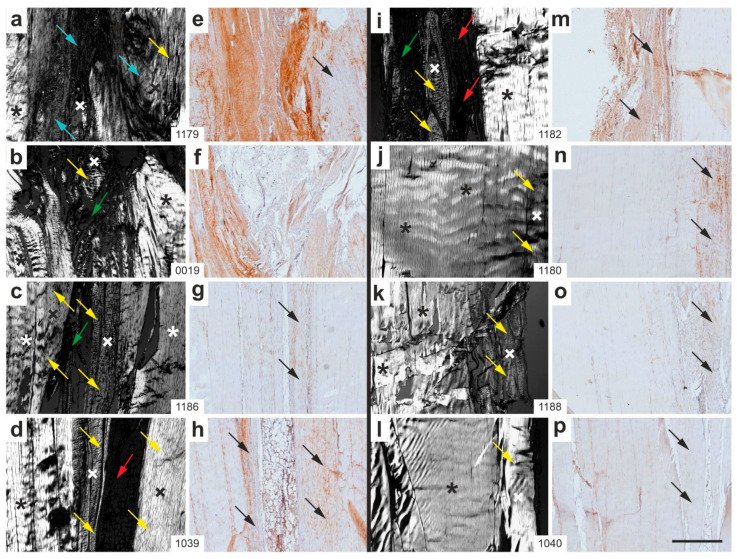
Representative polarization photomicrographs of 5 µm thick sections of the right common calcaneal tendon from all rabbits in Group 5 (UA-ADRCs/W12), stained with Safranin O/Fast Green at the site of surgery and treatment (**a**–**d**,**i**–**l**), along with corresponding immunohistochemical photomicrographs detecting aggrecan (**e**–**h**,**m**–**p**). The animal order (**a**/**e**,**b**/**f**,**c**/**g** … **l**/**p**) corresponds to that shown in Figure 10a (from left to right). Black and white asterisks denote original tendon tissue; Black and white crosses indicate newly formed connective tissue. Yellow arrows highlight newly formed connective tissue exhibiting the characteristic crimp pattern of tendons; blue arrows point to newly formed connective tissue lacking this crimp pattern; green arrows mark loose connective tissue and artifacts; red arrows indicate adipose tissue; and black arrows identify newly formed connective tissue with a crimp pattern in the immunohistochemical images corresponding to those seen in the polarization photomicrographs. Numbers in the white boxes linking each panel pair (**a**/**e**,**b**/**f**,**c**/**g** … **l**/**p**) indicate individual animal IDs according to the study protocol. The scale bar in panel (**p**) represents 500 µm and applies to all panels. Further details are provided in the text.

Figure 11 presents representative polarization photomicrographs of sections stained with Safranin O/Fast Green (Figure 11a–d,i–l) at the site of surgery and treatment, alongside corresponding photomicrographs of immunohistochemical detection of aggrecan (Figure 11e–h,m–p) from all rabbits in Group 5 (UA-ADRCs/W12).

**Figure 12:**
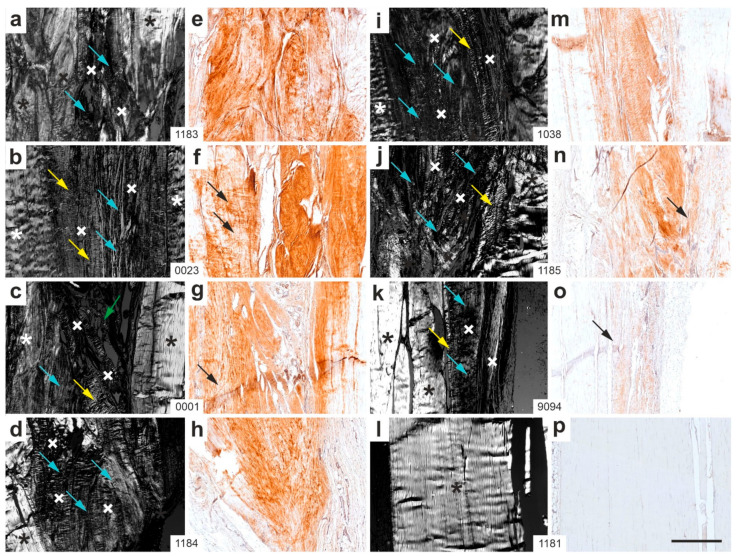
Representative polarization photomicrographs of 5 µm thick sections of the right common calcaneal tendon from all rabbits in Group 6 (sham/W12), stained with Safranin O/Fast Green at the site of surgery and sham treatment (**a**–**d**,**i**–**l**), along with corresponding immunohistochemical photomicrographs detecting aggrecan (**e**–**h**,**m**–**p**). The order of the animals (**a**/**e**,**b**/**f**,**c**/**g** … **l**/**p**) corresponds to that shown in Figure 10c (from left to right). Black and white asterisks mark original tendon tissue; black and white crosses indicate newly formed connective tissue. Yellow arrows highlight newly formed connective tissue displaying the characteristic crimp pattern of tendons; blue arrows indicate newly formed tissue lacking this pattern; green arrows point to loose connective tissue and artifacts; black arrows denote newly formed connective tissue in the immunohistochemical images that corresponds to tendon-like crimp structures observed in the polarization photomicrographs. Numbers in the white boxes connecting each pair of corresponding panels (**a**/**e**,**b**/**f**,**c**/**g** … **l**/**p**) indicate individual animal IDs according to the study protocol. The scale bar in panel (**p**) represents 500 µm and applies to all panels. Further details are provided in the text.

Figure 12 shows the corresponding photomicrographs from all rabbits in Group 6 (sham/W12).

The interpretation of Figure 11 and Figure 12 can be summarized as follows: (i) Tendon-like crimp in newly formed connective tissue (yellow arrows)—most sections from Group 5 (UA-ADRCs/W12) displayed well-developed crimp patterns in newly formed connective tissue, often integrated with or adjacent to original tendon tissue. The consistency across animals (Figure 11a,c,d,i,j,k,l) suggests widespread tendon-like remodeling. In contrast, crimped tissue in Group 6 (sham/W12) was less frequent and more disorganized, with newly formed regions often lacking the characteristic banded architecture of original tendon tissue or newly formed connective tissue. These findings indicate that UA-ADRC treatment promoted more frequent and structured crimp formation, consistent with enhanced tendon-like regeneration. (ii) Aggrecan expression in newly formed connective tissue with crimp pattern (black arrows)—in Group 5 sections, aggrecan expression was observed in multiple animals, typically co-localized with crimped regions (Figure 11e,g,h,m,n), suggesting active ECM production in structurally organized zones. In contrast, Group 6 sections showed weaker, more diffuse, or patchy aggrecan staining in fewer samples (Figure 12f,g,o), indicating less robust ECM remodeling and potentially less functional tissue repair. (iii) Non-crimped connective tissue (blue arrows)—in Group 5 sections, blue arrows marking non-crimped, newly formed connective tissue were relatively rare and scattered. By contrast, in Group 6 sections, such tissue was prevalent across numerous panels (Figure 12a–d,i–k), often dominating over crimped regions, which reflects the formation of structurally immature or disorganized connective tissue. (iv) Loose connective tissue (green arrows)—both groups exhibited occasional areas of loose connective tissue or artifacts, but these did not predominate in either group.

In summary, Group 6 (sham/W12) sections were characterized by less organized connective tissue formation, a predominance of non-crimped matrix, weaker and more inconsistent aggrecan expression in newly formed connective tissue, and fewer tendon-like features overall. In contrast, UA-ADRC treatment (Group 5) resulted in more structurally organized and molecularly active tendon regeneration.

### 2.8. Functional Histology and Functional Immunohistochemistry

The results presented in this section pertain to functional histology and functional immunohistochemistry, as the distal regions of the CCT under investigation did not include the sites of surgery, treatment or sham treatment.

**Figure 13:**
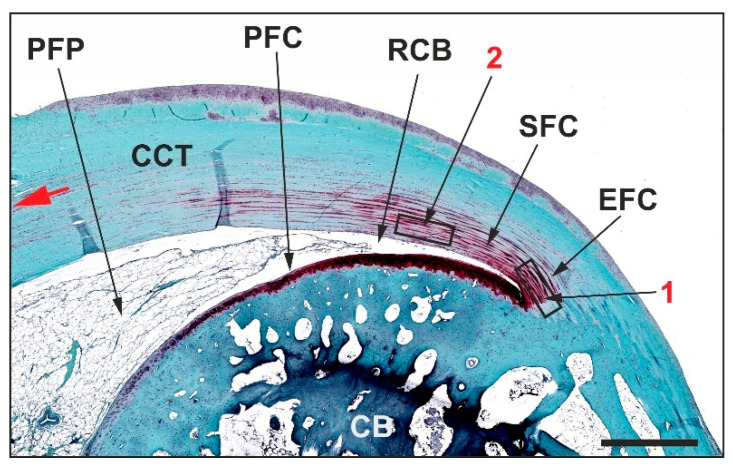
Anatomical details of the right common calcaneal tendon (CCT) at the site of calcaneal insertion in a representative photomicrograph of a 5 µm thick section stained with Safranin O/Fast Green from a rabbit in Group 5 (UA-ADRCs/W12). The red arrow denotes the longitudinal axis of the CCT. The scale bar represents 1 mm. Abbreviations: CB, calcaneal bone; CCT, common calcaneal tendon; EFC, enthesis fibrocartilage; PFC, periosteal fibrocartilage; PFP, precalcaneal fat pad; RCB, retrocalcaneal bursa; SFC, sesamoid fibrocartilage; 1, CCT at the site of enthesis fibrocartilage; 2, CCT at the site of sesamoid fibrocartilage.

Figure 13 shows anatomical details of the distal right CCT at its insertion on the calcaneus, depicted in a representative 5 µm thick section stained with Safranin O/Fast Green from a rabbit in Group 5 (UA-ADRCs/W12).

**Figure 14:**
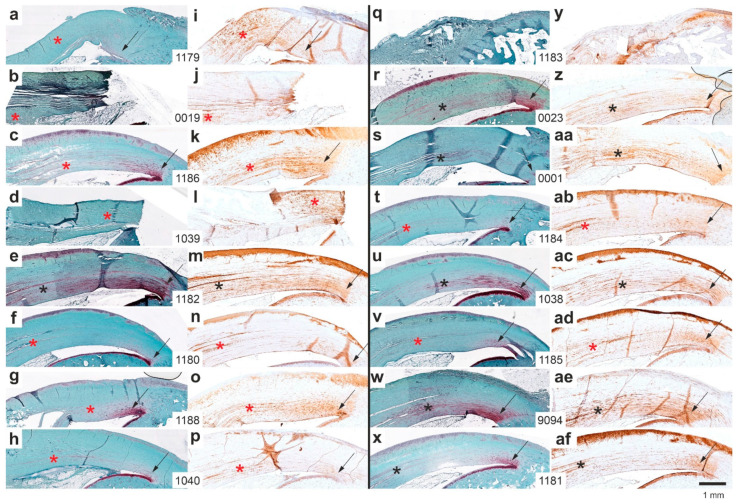
Representative low-magnification photomicrographs of 5 µm thick sections of the right (surgery, treatment or sham treatment) distal common calcaneal tendon (CCT) at the site of calcaneal insertion (comparable to Figure 13), stained with Safranin O/Fast Green (**a**–**h**,**q**–**x**) or processed for immunohistochemical detection of aggrecan (**i**–**p**,**y**–**af**) from all rabbits in Group 5 (UA-ADRCs/W12) (**a**–**p**) and Group 6 (sham/W12) (**q**–**af**). The order of the animals (**a**/**i**,**b**/**j**,**c**/**k** … **x**/**af**) matches that shown in Figure 10a,c (left to right). Arrows mark the transition from the CCT to the calcaneus. Asterisks indicate additional aggrecan immunolabeling in the CCT at sites corresponding to the formation of sesamoid fibrocartilage, as well as the respective regions in the Safranin O/Fast Green-stained sections (black and red asterisks are defined in the main text). Numbers in the white boxes linking each pair of corresponding panels (**a**/**i**,**b**/**j**,**c**/**k** … **x**/**af**) indicate individual animal IDs according to the study protocol. The scale bar in panel (**af**) represents 1 mm and applies to all panels. Further details are provided in the text.

Figure 14 presents representative low-magnification photomicrographs of 5 µm thick sections of the distal right CCT at the calcaneal insertion site, stained either with Safranin O/Fast Green (Figure 14a–h,q–x) or processed for immunohistochemical detection of aggrecan (Figure 14i–p,y–af), from all rabbits in Group 5 (UA-ADRCs/W12) (Figure 14a–p) and Group 6 (sham/W12) (Figure 14q–af).

In two rabbits—rabbit 0019 (Figure 14b,j) and rabbit 1039 (Figure 14d,l)—the CCT was torn from the calcaneus. Additionally, in rabbit 1183 (Figure 14q,y), the original CCT was not attached to the calcaneus, but newly formed connective tissue was. Notably, despite firm attachment, this tissue showed no aggrecan immunolabeling, suggesting possible functional inactivity.

Asterisks in the panels indicate additional aggrecan immunolabeling at the site of sesamoid fibrocartilage formation (Figure 14i–p,z–af), with corresponding regions highlighted in the Safranin O/Fast Green-stained sections (Figure 14a–h,r–x). One rabbit in Group 5 (rabbit 1182, Figure 14e,m) and five rabbits in Group 6 (rabbits 0023 [Figure 14r,z], 0001 [Figure 14s,aa], 1038 [Figure 14u,ac], 9094 [Figure 14w,ae], and 1181 [Figure 14x,af]) exhibited clear Safranin O staining at the sites of aggrecan immunolabeling (black asterisks). Conversely, seven rabbits in Group 5 (all except rabbit 1182) and only two rabbits in Group 6 (rabbits 1184 [Figure 14t,ab] and 1185 [Figure 14v,ad]) showed little or no Safranin O staining at these sites (red asterisks in Figure 14). This difference was statistically significant (Fisher’s exact test; *p* = 0.041).

**Figure 15:**
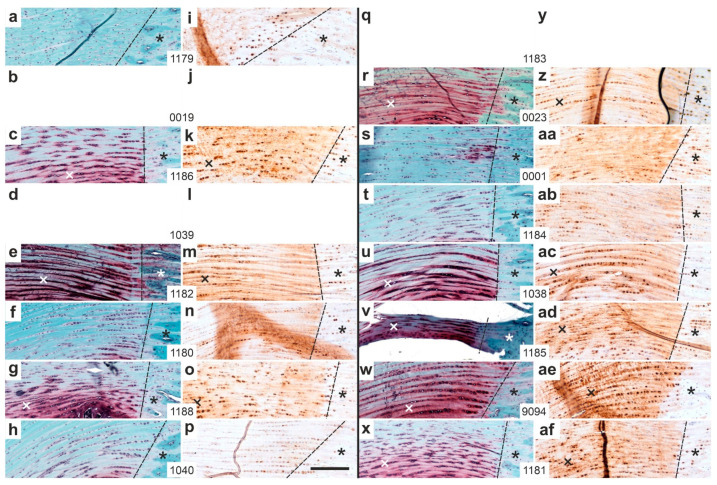
Representative high-magnification photomicrographs of 5 µm thick sections of the right (surgery, treatment or sham treatment) distal common calcaneal tendon (CCT) at the site of enthesis fibrocartilage (cf. rectangle 1 in Figure 13), stained with Safranin O/Fast Green (**a**–**h**,**r**–**x**) or processed for immunohistochemical detection of aggrecan (**i**–**p**,**z**–**af**) from all rabbits in Group 5 (UA-ADRCs/W12) (**a**–**p**) and Group 6 (sham/W12) (**r**–**af**). The order of the animals (**a**/**i**,**b**/**j**,**c**/**k** … **x**/**af**) corresponds to Figure 10a,c (from left to right). No photomicrographs are shown for the right CCT of rabbits 0019 (**b**,**j**) and 1039 (**d**,**l**) due to tendon detachment from the calcaneus (cf. Figure 14b,j,d,l). Likewise, no images are shown for rabbit 1183 (**q**,**y**), as the structure at the insertion site was not the original CCT but newly formed connective tissue (cf. Figure 14q,y). Dashed lines indicate the transition from the CCT to the calcaneus (marked by black or white asterisks), Black and white crosses highlight regions of intense Safranin O staining in the enthesis fibrocartilage. Numbers in the white boxes linking each pair of corresponding panels (**a/i**,**b/j**,**c/k** … **x/af**) indicate individual animal IDs according to the study protocol. The scale bar in panel (**p**) represents 200 µm and applies to all panels. Further details are provided in the text.

Figure 15 shows representative high-magnification photomicrographs of 5 µm thick sections from the distal right CCT at the site of enthesis fibrocartilage (cf. rectangle 1 in Figure 13), stained with Safranin O/Fast Green (Figure 15a–h,r–x) or processed for aggrecan immunolabeling (Figure 15i–p,z–af), from all rabbits in Group 5 (ADRCs/W12; Figure 15a–p) and Group 6 (sham/W12; Figure 15r–af).

In both groups, some rabbits showed pronounced Safranin O staining and strong intracellular, but weaker extracellular, aggrecan immunolabeling (Figure 15c,k,e,m,g,o,r,z,u,ac,v,ad,w,ae,x,af). Others displayed only faint Safranin O staining along with weak intra- and extracellular aggrecan labeling (Figure 15a,i,f,n,h,p,s,aa,t,ab). Based on these images, no clear differences could be identified between the two groups.

**Figure 16:**
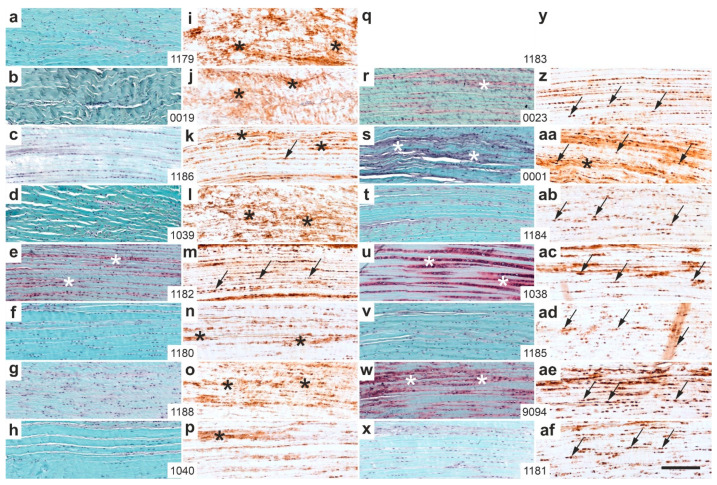
Representative high-magnification photomicrographs of 5 µm thick sections of the right (surgery, treatment or sham treatment) distal common calcaneal tendon (CCT) at the site of sesamoid fibrocartilage (cf. rectangle 2 in Figure 13), stained with Safranin O/Fast Green (**a**–**h**,**r**–**x**) or processed for immunohistochemical detection of aggrecan (**i**–**p**,**z**–**af**) from all rabbits in Group 5 (UA-ADRCs/W12) (**a**–**p**) and Group 6 (sham/W12) (**r**–**af**). The order of the animals (**a**/**i**,**b**/**j**,**c**/**k** … **x**/**af**) corresponds to Figure 10a,c (from left to right). No photomicrographs are shown for the right CCT of rabbit 1183 (**q**,**y**), as the tissue present at the insertion site was not the original tendon but newly formed connective tissue (cf. Figure 14q,y). Arrows indicate intracellular immunolabeling for aggrecan; black asterisks denote extracellular aggrecan immunolabeling; white asterisks highlight areas of intense Safranin O staining corresponding to intracellular aggrecan expression. Numbers in the white boxes linking each pair of corresponding panels (**a/i**,**b/j**,**c/k** … **x/af**) indicate individual animal IDs according to the study protocol. The scale bar in panel (**af**) represents 200 µm and applies to all panels. Further details are provided in the text.

Figure 16 presents representative high-magnification photomicrographs of 5 µm thick sections from the distal right CCT at the site of sesamoid fibrocartilage (cf. rectangle 2 in Figure 13), stained with Safranin O/Fast Green (Figure 16a–h,r–x) or processed for aggrecan immunolabeling (Figure 16i–p,z–af), again from all rabbits in Group 5 (UA-ADRCs/W12; Figure 16a–p) and Group 6 (sham/W12; Figure 16r–af).

Two rabbits in Group 5 (UA-ADRCs/W12) (rabbits 1186 [Figure 16k] and 1182 [Figure 16m]) and all rabbits in Group 6 (sham/W12) exhibited intracellular aggrecan immunolabeling at the site of sesamoid fibrocartilage (indicated by arrows in Figure 16). In contrast, all rabbits in Group 5 except 1182 (Figure 16m), and only one rabbit in Group 6 (rabbit 0001, Figure 16aa), showed intense extracellular aggrecan immunolabeling (black asterisks in Figure 16). These differences were statistically significant (Fisher’s exact test; *p* = 0.007 for intracellular labeling and *p* = 0.010 for extracellular labeling).

Additionally, the distal CCT of one rabbit in Group 5 (rabbit 1182; Figure 16e) and four rabbits in Group 6 (rabbits 0012 [Figure 16r], 0001 [Figure 16s], 1038 [Figure 16u] and 9094 [Figure 16w]) showed intense Safranin O staining at sites corresponding to intracellular aggrecan labeling (white asterisks in Figure 16).

**Figure 17:**
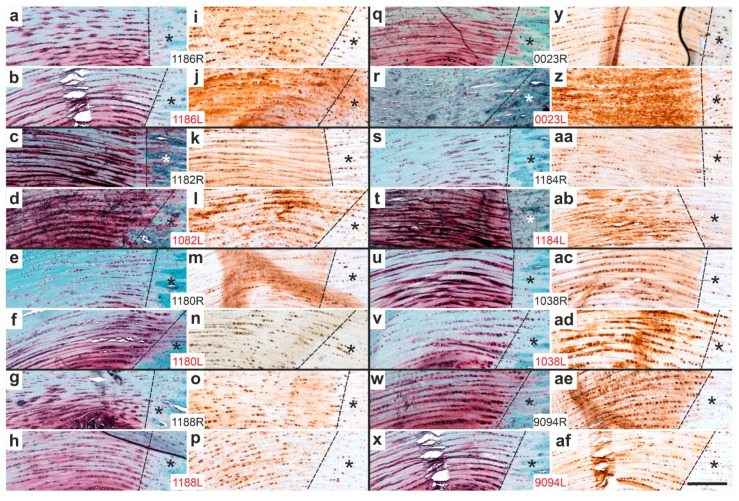
Representative high-magnification photomicrographs of 5 µm thick sections of the distal common calcaneal tendon (CCT) at the site of enthesis fibrocartilage (cf. rectangle 1 in Figure 13), stained with Safranin O/Fast Green (**a**–**h**,**q**–**x**) or processed for immunohistochemical detection of aggrecan (**i**–**p**,**y**–**af**), from four rabbits each in Group 5 (UA-ADRCs/W12) (**a**–**p**) and Group 6 (sham/W12) (**q**–**af**). Sections were taken from the right CCT (surgery, treatment or sham treatment side) or the left CCT (no surgery, no treatment or sham treatment side). Dashed lines indicate the transition from the CCT to the calcaneus, marked by black and white asterisks. Numbers in the white boxes linking each pair of corresponding panels (**a**/**i**,**b**/**j**,**c**/**k** … **x**/**af**) indicate individual animal IDs according to the study protocol. The scale bar in panel (**af**) represents 200 µm and applies to all panels. Further details are provided in the text.

Figure 17 shows representative high-magnification photomicrographs of 5 µm thick sections from the right and left distal CCTs at the site of enthesis fibrocartilage (cf. rectangle 1 in Figure 13), stained with Safranin O/Fast Green (Figure 17a–h,q–x) or processed for aggrecan immunohistochemistry (Figure 17i–p,y–af), from four rabbits each in Group 5 (ADRCs/W12; Figure 17a–p) and Group 6 (sham/W12; Figure 17q–af).

**Figure 18:**
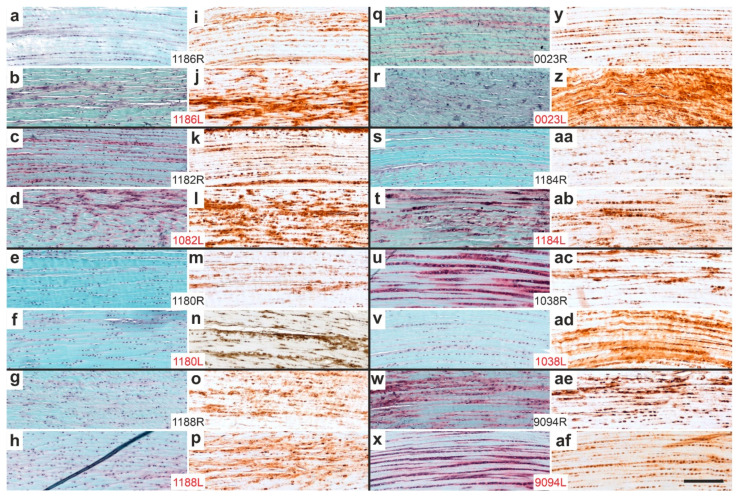
Representative high-magnification photomicrographs of 5 µm thick sections of the distal common calcaneal tendon (CCT) at the site of sesamoid fibrocartilage (cf. rectangle 2 in Figure 13), stained with Safranin O/Fast Green (**a**–**h**,**q**–**x**) or processed for immunohistochemical detection of aggrecan (**i**–**p**,**y**–**af**), from four rabbits each in Group 5 (UA-ADRCs/W12) (**a**–**p**) and Group 6 (sham/W12) (**q**–**af**). Sections were obtained from the right CCT (surgery, treatment or sham treatment side) or the left CCT (no surgery, no treatment or sham treatment side). Numbers in the white boxes linking each pair of corresponding panels (**a**/**i**,**b**/**j**,**c**/**k** … **x**/**af**) indicate individual animal IDs according to the study protocol. The scale bar in panel (**af**) represents 200 µm and applies to all panels. Further details are provided in the text.

Figure 18 presents the corresponding high-magnification photomicrographs of 5 µm thick sections at the site of sesamoid fibrocartilage (cf. rectangle 2 in Figure 13), stained with Safranin O/Fast Green (Figure 18a–h,q–x) or processed for aggrecan detection (Figure 18i–p,y–af), also from four rabbits each in Group 5 (ADRCs/W12; Figure 18a–p) and Group 6 (sham/W12; Figure 18q–af).

Figure 17 and Figure 18 illustrate inter-individual variability in both Safranin O/Fast Green staining and aggrecan immunolabeling patterns in rabbits from Groups 5 (UA-ADRCs/W12) and 6 (sham/W12). These differences may partly reflect subtle anatomical and biomechanical variations in the CCT and calcaneus—specifically, the degree to which the CCT wraps around the calcaneus, which affects the magnitude and distribution of lateral compressive forces. Notably, intra-individual differences between the left and right CCTs were more pronounced in Group 6 than in Group 5.

In summary, UA-ADRC treatment (Group 5) led to more functionally organized and structurally mature regeneration at the distal CCT compared to sham treatment (Group 6). Safranin O/Fast Green staining and aggrecan immunolabeling revealed that Group 5 rabbits showed more frequent and pronounced formation of sesamoid and enthesis fibrocartilage, as well as stronger extracellular aggrecan expression. This was in line with the finding that tendon-like crimp patterns were more consistently observed in Group 5 (Figure 11), whereas Group 6 sections displayed predominantly non-crimped, disorganized connective tissue (Figure 12). Intracellular aggrecan labeling—indicative of early fibrocartilaginous activity—was more common in Group 6, while extracellular labeling—reflecting mature matrix deposition—was more frequent in Group 5 (Figure 15 and Figure 16). Statistical analysis confirmed these differences, showing significant group-wise variation in Safranin O staining and aggrecan localization (Figure 14 and Figure 16). Collectively, these findings indicate that UA-ADRC treatment promotes more advanced and spatially coordinated fibrocartilage formation and tendon-to-bone integration, key elements of functional tendon regeneration.

### 2.9. Design-Based Stereologic Analysis

**Figure 19:**
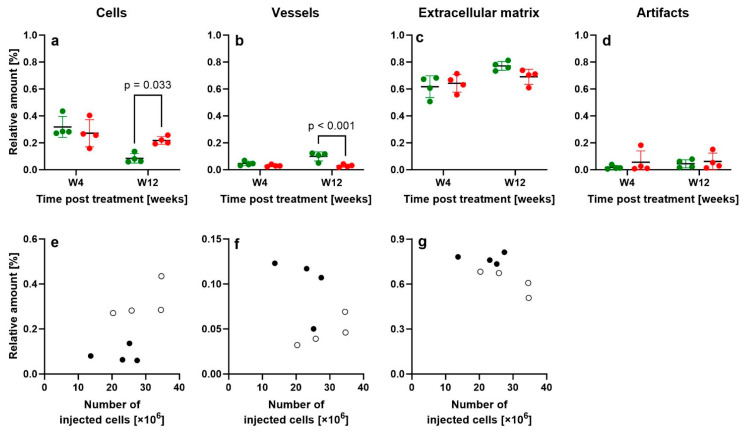
Scatter dot plots with mean ± standard deviation (horizontal lines) showing the relative amount of (**a**) cells, (**b**) vessels, (**c**) extracellular matrix and (**d**) artifacts in the newly formed connective tissue of the right common calcaneal tendon (surgery, treatment or sham treatment side) in rabbits from Group 1 (UA-ADRCs/W4; green dots at W4 in (**a**–**d**); white dots in (**e**–**g**)), Group 3 (sham/W4; red dots at W4 in (**a**–**d**)), Group 2 (UA-ADRCs/W12; green dots at W12 in (**a**–**d**); black dots in (**e**–**g**)) and Group 4 (sham/W12; red dots at W12 in (**a**–**d**)). Results of Bonferroni’s multiple comparison test with *p* < 0.05 are indicated in panels (**a**,**b**). Further details are provided in the text.

Figure 19 presents the results of design-based stereologic analysis (c.f. [20]) quantifying the relative amounts of cells, vessels, ECM and artifacts in the newly formed connective tissue from rabbits in Groups 1–4.

The outcomes of the two-way ANOVA (factors: treatment type and time point), as well as the 95% confidence intervals of mean differences at W4 and W12, are summarized in Table 1, while Bonferroni’s multiple comparison test results for pairwise comparisons are shown in Figure 19a–d.

No significant differences were observed between Group 1 (UA-ADRCs/W4) and Group 3 (sham/W4). However, rabbits in Group 2 (UA-ADRCs/W12) exhibited a significantly lower relative amount of cells and a significantly higher relative amount of vessels in the newly formed connective tissue compared to Group 4 (sham/W12) (*p* < 0.05).

Notably, in both Group 1 and Group 2, there was no significant correlation between the initial cell dose and the relative amounts of cells, vessels or ECM in the newly formed tissue (cf. Figure 19e–g).

### 2.10. Non-Destructive Biomechanical Analysis

**Figure 20:**
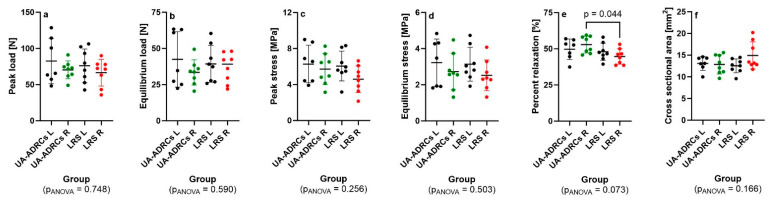
Scatter dot plots with mean ± standard deviation (horizontal lines) showing (**a**) peak load, (**b**) equilibrium load, (**c**) peak stress, (**d**) equilibrium stress, (**e**) percent relaxation, and (**f**) cross-sectional area of the common calcaneal tendon (CCT) in rabbits from Group 5 (UA-ADRCs/W12) and Group 6 (sham/W12). Data for the left (intact/untreated) CCT are shown as black dots in panels (**a**–**f**); data for the right (surgery/treatment side) CCT are shown as green dots for Group 5 and red dots for Group 6. Results of one-way ANOVA are indicated at the bottom of each panel. Results of Bonferroni’s multiple comparison test with *p* < 0.05 are shown in panel (**e**). In this panel, the 95% confidence interval of the mean difference between the Groups UA-ADRCs R and RLS R was 0.161 to 16.3. Further details are provided in the text.

Figure 20a–e present the results of the non-destructive biomechanical analysis of the left and right CCTs from rabbits in Group 5 (UA-ADRCs/W12) and Group 6 (sham/W12); Figure 20f displays the corresponding measurements of the cross-sectional area of these tendons.

Table 2 summarizes the effect size (Cohen’s f) and observed power from the one-way ANOVA of the data in Figure 20, along with the total and per-group number of rabbits required to achieve 80% power for the same effect size across four groups.

Statistical analysis revealed a significantly lower mean percent relaxation in the right (injured/sham-treated) CCTs of Group 6 (ADRCs/W12) compared to the right (injured/treated) CCTs of Group 5 (sham/W12; Figure 20e). All other measurements would have required to investigate more rabbits (between 11 and 39 per group) to achieve 80% power with the same effect sizes.

**Figure 21:**
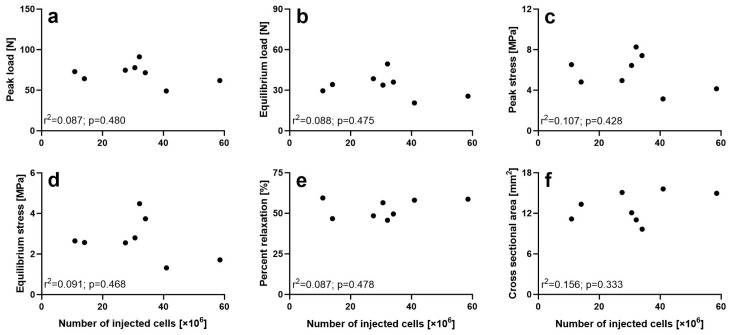
Scatter dot plots and linear regression analyses showing (**a**) peak load, (**b**) equilibrium load, (**c**) peak stress, (**d**) equilibrium stress, (**e**) percent relaxation, and (**f**) cross-sectional area of the right common calcaneal tendon in rabbits from Group 5 (UA-ADRCs/W12) as a function of the number of injected cells. Results of linear regression analysis, including the coefficient of determination (r^2^) and *p*-value, are provided in each panel. Further details are provided in the text.

There were no significant correlations between the results of the biomechanical analysis and the initial cell dose, as shown in Figure 21a–f.

## 3. Discussion

UA-ADRCs cannot be labeled and are therefore undetectable in host tissue [21,22]. Additionally, their molecular and cellular mechanisms cannot be investigated in vitro, as their composition changes immediately after plating for culture, resulting in adipose-derived stem cells (ADSCs) [23,24]. ADSCs, well-studied in the literature [25,26,27], can differentiate into somatic cells of all three germ layers [3,22] and can be detected in vivo after labeling with fluorescent markers (e.g., GFP in [21,28]). However, ADSCs represent only a minor fraction of UA-ADRCs [5]. Consequently, conclusions on UA-ADRCs’ effects must be drawn from observed structural and functional tissue changes post-treatment compared to sham or alternative treatments.

To our knowledge, this is the first study to comprehensively evaluate the histological, immunohistochemical and biomechanical characteristics of tendons after partial-thickness injury and UA-ADRC treatment in vivo. The rationale for applying multiple staining and imaging methods includes the following: (i) tendons consist of crimped, longitudinally aligned type I collagen fibrils, which function as shock absorbers during loading [29,30]; (ii) type I procollagen forms extracellularly into cross-linked fibrils [31,32]; (iii) type III collagen, typical of scar tissue, is less organized and mechanically inferior to type I collagen [33,34]; (iv) CD163 marks M2 macrophages, associated with anti-inflammatory activity [35,36], which is relevant in tendon healing and inflammation [37,38,39]; (v) pro-inflammatory cytokines (e.g., IL-1β, TNF-α) impair tenogenic factors like scleraxis [40,41,42]; (v) aggrecan, a marker of fibrocartilage, increases tissue water content and compression resistance [43,44]; and (vi) Safranin O stains proteoglycans red, indicating water-storing capacity [45,46], whereas fast green stains collagen.

Our findings show that UA-ADRC treatment led to seamless integration of new tendon-like connective tissue with improved biomechanical function, whereas sham-treated tendons showed disorganized, scar-like tissue, vascular and adipose tissue formation at the junction, and impaired biomechanical function. Specifically, UA-ADRC treatment resulted in significantly higher percent relaxation than sham treatment at W12 post-treatment (Figure 20e). In non-destructive biomechanical analysis of tendons, percent relaxation is a measure of the tendon’s viscoelastic behavior—specifically, how much the tendon “relaxes” (reduces internal stress) over time under a constant strain. It is a sensitive marker of tissue quality and functional maturity. Percent relaxation is defined as the percentage decrease in stress from the initial peak stress to the equilibrium stress during a stress-relaxation test (i.e., when the tendon is held at a fixed length) [47]. Higher percent relaxation (observed after UA-ADRC treatment) indicates greater viscoelasticity, meaning the tendon tissue is more capable of dissipating stress over time [47]—this is typical of healthy or well-integrated tissue. In contrast, lower percent relaxation may suggest stiffer or scar-like tissue, which lacks normal viscoelastic damping properties [47] and could be more prone to re-injury. In summary, increased relaxation after treatment (as with UA-ADRCs) indicates the formation of tissue that better reproduces the mechanical behavior of native tendon.

Four main findings emerge from Figure 14, Figure 15, Figure 16, Figure 17 and Figure 18: (i) Aggrecan expression was localized to newly formed connective tissue lacking the typical crimp pattern, likely reflecting adaptation to compressive stress [44,45]. (ii) Intracellular aggrecan, unlike extracellular proteoglycans (Safranin O positive), does not contribute to ECM water retention. This pattern was more frequent in Group 6 (sham/W12) sections than in Group 5 (UA-ADRCs/W12) sections. (iii) Reduced aggrecan expression in Group 6 sections may indicate delayed functional recovery. (iv) The injured/treatment-side CCT in Group 5 sections showed signs of functional restoration at 12 weeks, unlike Group 6 sections.

A significant reduction in cell density in UA-ADRC-treated tendons from W4 to W12 (–73%) compared to sham treatment (–20%) (Figure 19a) parallels findings in developmental studies [29,48], suggesting that UA-ADRC-induced regeneration mimics late fetal/early postnatal maturation.

Another key observation was adipose tissue formation in the defect site after sham but not UA-ADRC treatment (Figure 4). A similar finding was noted in a clinical case report of bone regeneration using a combination of UA-ADRCs, plasma rich in grow factors (PRGF-2) and an osteoinductive scaffold (OIS) vs. PRGF-2/OIS alone, with lower adipose tissue formation after treatment with UA-ADRCs [49]. This underscores that ADRCs are not simply “fat stem cells” but include vascular wall-derived stem cells [3,21,22].

Comparable histological patterns were seen in a supraspinatus tendon biopsy 10 weeks post–UA-ADRC treatment, showing organized connective tissue with crimp, type I procollagen and tenocyte markers, but no adipocytes [11].

Only two studies have evaluated UA-ADRCs in experimental tendon injury [50,51], showing increased type I collagen, better collagen I/III ratio and improved biomechanics. While different in design, these findings are consistent with our results. In contrast, treatment with microfragmented fat improved gene expression but not mechanical properties in a sheep model [52], highlighting differences between fat-derived materials and UA-ADRCs [5,53].

A clinical study found no benefit of UA-ADRCs injected into bone–tendon–bone grafts during anterior cruciate ligament reconstruction [54]. This may reflect suboptimal cell delivery, possibly limited to tendon regions. Studies on cultured autologous ADSCs (Table 3; [7,55,56,57,58,59,60,61,62,63,64,65,66,67,68,69,70]) reported positive effects but differed significantly in design, focusing on surgical repair augmentation.

Collectively, these studies lack evidence for seamless tissue integration, prevention of vascular/adipose tissue formation at junctions, or substantial cell reduction over time, as found in this study after treatment with UA-ADRCs.

Recent studies of allogeneic or xenogenic ADSCs [71,72,73] highlight potential industrial scalability and consistent quality [74,75,76], but clinical data are less convincing. No biomechanical benefit was seen in several models [76,77,78]. Thus, the theoretical advantages of allogeneic ADSCs remain unproven in practice.

The use of allogeneic ADRCs, as proposed in [79,80,81], improved biomechanics in a rabbit model, but histological assessment was limited. Moreover, xenogenic ADRC use has been linked to chronic inflammation and fibrosis [82], making this approach clinically questionable [83].

A limitation of the present study is the inability to determine UA-ADRCs’ mechanisms of action, as they cannot be labeled (in line with clinical application [1,2,6,11,49]) or cultured in native composition. These effects likely result from complex in vivo interactions, which are not reproducible in vitro.

Four possible main mechanisms of action of UA-ADRCs in tendon regeneration are discussed (see also [84]): (1) UA-ADRCs secrete SDF-1α, TGF-β1, and IGF-1—higher than in cultured ADSCs—and these are also released by M2 macrophages [85,86,87,88]. Mesenchymal stem cells contained in UA-ADRCs (UA-ADRC MSCs) may also convert M1 to M2 macrophages [89,90]. (2) UA-ADRC MSCs may differentiate into tenocytes and integrate into host tissue, as shown for cultured ADSCs in animal models [25,61] and in vitro [91]. (3) Human type I collagen was detected in rat tendons treated with human cultured ADSCs [92], suggesting donor cell contribution to ECM formation. (4) Exosomes released by UA-ADRCs may modulate the expression of extracellular matrix-related genes, promote macrophage polarization and deliver therapeutic microRNAs to the site of injury [93,94].

Clinically, identifying exact mechanisms is secondary. Optimizing ADRCs by manipulation would reclassify them as ATMPs under EMA guidelines [95] or violate the “minimally manipulated” definition under FDA regulations (21 CFR 1271.10(a)) [96,97,98]. Clinically relevant is the composition of UA-ADRCs—rich in MSCs, endothelial progenitor cells and M2 macrophages. The isolation technology used here yields the highest relative numbers of these cell types, independent of donor characteristics [5].

A minor limitation is the absence of detailed characterization of UA-ADRCs isolated from rabbit adipose tissue using the Transpose RT system (InGeneron). Nevertheless, the exact composition of the injected UA-ADRCs is not critical for this study. Literature data show that human UA-ADRCs isolated with the same system exhibit higher cell viability and a greater proportion of regenerative cells compared to other commercially available enzymatic isolation systems (Table 4).

A further limitation is the use of an acute tendon defect model, whereas most partial-thickness tears occur in tendinopathic tendons [101,102,103]. However, the ideal tendinopathy model is still lacking [104]. Models must match the therapy: platelet rich plasma (PRP) or exosome therapies, which stimulate resident cells, would not be well-suited to this model [105,106,107,108,109,110]. PRP has also shown limited clinical value in RCTs for rotator cuff disease [111,112]. UA-ADRCs, by contrast, target injuries where local stem cell reserves are insufficient [24], making this defect model appropriate. Moreover, structural regeneration is the critical outcome, as clinical endpoints such as pain and mobility are subjective. Since biopsy is not feasible in human application, imaging must substitute. Thus, this study provides evidence of true structural regeneration—suggesting that UA-ADRC therapy could be genuinely structure-modifying, not merely symptom-modifying [113].

## 4. Materials and Methods

This study was approved by the Colorado State University (CSU) Institutional Animal Care and Use Committee (Fort Collins, CO, USA) (Protocol #1473; approval issued on 1 February 2021 and renewed/amended on 1 February 2022).

A total of 32 skeletally mature female New Zealand white rabbits (Oryctolagus cuniculus), aged 9–13 months and weighing 4.8 ± 0.6 kg (mean ± SD; range, 4.1–6.2 kg), were used. The animals were sourced from Western Oregon Rabbit Co. (Philomath, OR, USA) and housed under standard conditions at the CSU Laboratory Animal Resources Building (Fort Collins, CO, USA), with 12 h light/dark cycles, a stable temperature of approximately 25 °C, and individual housing in standard rabbit cages on slatted floors with daily cleaning and bi-weekly cage changes. The rabbits received commercial rabbit chow (Envigo Teklad 2031; Envigo, Indianapolis, IN, USA) and grass hay mix ad libitum, along with unrestricted access to tap water. All animals were naïve, had not participated in any prior studies, and were subjected to a veterinarian-administered physical examination before the study commenced. Only animals with acceptable health status, weight and age were included. Body weight was recorded bi-weekly throughout the study.

In the first cohort, 16 rabbits were randomly assigned to four experimental groups (Groups 1–4; n = 4 per group) by drawing lots. The second cohort was similarly randomized into two groups (Groups 5 and 6; n = 8 per group; Table 5).

The chosen sample sizes reflected a reasonable balance between the ethical imperative to minimize animal use and the extensive experience of the colleagues at the Preclinical Surgical Research Laboratory (K.S., L.B., J.E. and H.S.) as well as the Orthopaedic Bioengineering Research Laboratory, Department of Mechanical Engineering (C.P., B.G. and K.L.) at Colorado State University (Fort Collins, CO, USA) in detecting statistically significant effects in comparable rabbit studies. No animals were excluded after enrollment, and all collected data were fully reported in the results.

While researchers were not blinded to the treatment allocation, histopathological slide evaluations were conducted in a blinded manner.

For adipose tissue harvesting, rabbits were placed in sternal recumbency, while right gastrocnemius tendon defect production required left lateral recumbency. General anesthesia was induced and monitored at five-minute intervals using a combination of Buprenorphine (0.03 mg/kg BW; Par Pharmaceutical, Chestnut Ridge, NY, USA), Glycopyrrolate (0.005 mg/kg BW; Somerset Therapeutics, Hollywood, FL, USA), Ketamine (25 mg/kg BW; Dechra Veterinary Products, Overland Park, KS, USA), Dexmedetomidine (0.02 mg/kg BW; Dechra Veterinary Products) and Isoflurane inhalation (1–5%; VetOne, Boise, ID, USA) during surgery. Vital signs were monitored throughout, including heart rate, respiratory rate, ECG, pulse oximetry, CO_2_ exhalation and reflex responses.

For rabbits in Groups 1, 2 and 5, the interscapular region was shaved, disinfected, and a 2 cm incision made to access the fat depot (c.f. [114]). Approximately 21.1 ± 5.3 g of adipose tissue (range, 11.6–30.5 g) was harvested, immediately stored in sterile RLS, and processed using the Transpose RT system (InGeneron) under sterile conditions. The average total cell yield was 14.5 × 10^5^ ± 7.1 × 10^5^ cells/g of adipose tissue, with a mean cell viability of 82.6 ± 5.4%. The average live cell yield was 11.8 × 10^5^ ± 5.3 × 10^5^ cells/g. Each batch was processed individually to prevent cross-contamination, and was assigned a unique lot number.

**Figure 22:**
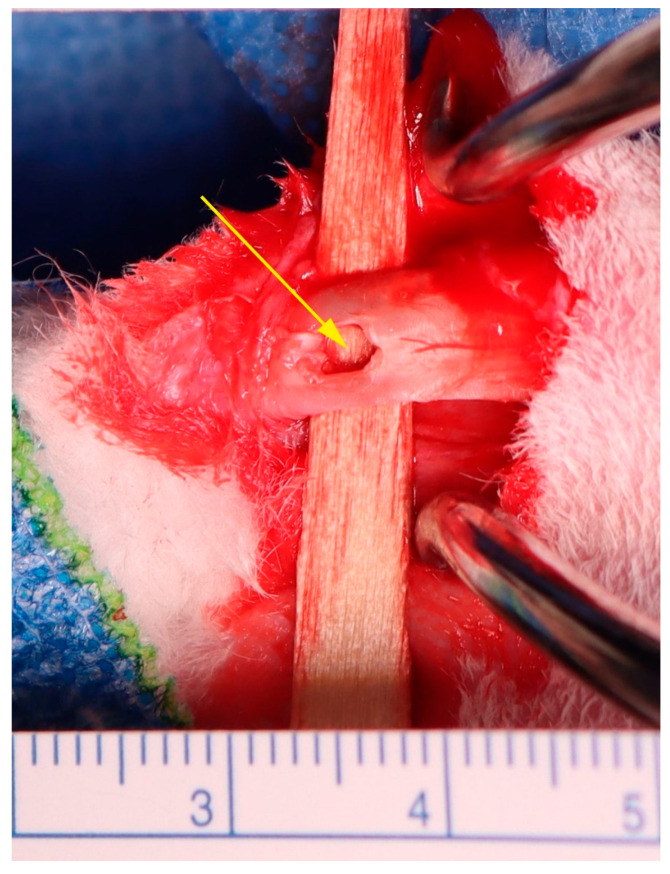
Production of a full-thickness defect in the midsubstance of the rabbit gastrocnemius tendon. The defect (yellow arrow) was produced with a punch with a diameter of 3 mm, approximately 2.5 cm from the calcaneus insertion.

Surgical procedures involved shaving and disinfecting the right hindlimb, followed by a 1–3 cm posterior incision to access the CCT (c.f. [115]). The peritenon was dissected, and a 3 mm full-thickness defect was produced in the gastrocnemius tendon approximately 2.5 cm from the calcaneal insertion (Figure 22; c.f. [116]).

The peritenon was partially closed before injection. Rabbits in Groups 1, 2, and 5 received an injection of 28.3 × 10^6^ ± 11.6 × 10^6^ UA-ADRCs (in 0.5 mL RLS or saline) into and around the defect site. Groups 3 and 4 received 0.5 mL RLS, and Group 6 received 0.5 mL saline. The peritenon and skin were then fully closed. The contralateral CCT was left untouched. Postoperative analgesia included subcutaneous Buprenorphine (0.03 mg/kg BW; Par Pharmaceutical) at 12 and 24 h, and Meloxicam (1 mg/kg BW; VetOne) administered daily for up to 7 days.

Euthanasia was performed under deep anesthesia using Ketamine (5–30 mg/kg BW; Dechra Veterinary Products) and Dexamedetomidine (0.05–0.125 mg/kg BW; Dechra Veterinary Products) or Xylazine (5 mg/kg BW; VetOne), followed by Isoflurane (VetOne) inhalation and intravenous Pentobarbitone sodium (88 mg/kg BW; Dechra Veterinary Products). This was carried out in accordance with American Veterinary Medical Association guidelines. Rabbits in Groups 1 and 3 were euthanized at 4 weeks post-treatment, while those in Groups 2, 4, 5, and 6 were euthanized at 12 weeks.

Both hindlimbs were collected en bloc. The CCT and surrounding treatment regions were dissected and isolated. Tendon segments from Groups 1–4 were sectioned into approximately 3 cm blocks centered around the defect. For Groups 5 and 6, the calcaneal insertion was preserved, and samples underwent decalcification in 5% formic acid prior to further processing. All tissues were fixed in 10% neutral buffered formalin, embedded in paraffin and sectioned longitudinally at 5 µm. Three sections per limb were stained with Picrosirius Red, Hematoxylin and Eosin (H&E), or Safranin O/Fast Green. Sections were shipped to the Institute of Anatomy, Faculty of Medicine, LMU Munich (Munich, Germany) for further analysis.

Immunohistochemistry was performed at LMU Munich on deparaffinized, rehydrated sections. After pre-treatment and blocking, sections were incubated with primary antibodies targeting procollagen 1, type III collagen, CD163 and aggrecan (Table 6).

Detection was achieved with the Vectastain Elite ABC Kit (Vector Laboratories, Burlingame, CA, USA) and diaminobenzidine chromogen (Vector Impact DAB chromogen solution; Vector Laboratories), followed by hematoxylin counterstaining. Negative controls used phosphate-buffered saline (PBS) instead of primary antibodies. Microscopic evaluation was conducted by C.S., C.A., S.M., and E.A.

Design-based stereologic analysis was used to quantify cells, vessels and matrix areas in the newly formed connective tissue adjacent to the tendon. This was performed using point-counting methods (c.f. [20]) with ~501 points per section, using a computerized stereology workstation, consisting of a modified light microscope (Axioskop; Carl Zeiss Microscopy, Jena, Germany) with Plan-Neofluar objectives 1.25× (numerical aperture [NA] = 0.03), 2.5× (NA = 0.085), Plan-Apochromat objectives 5× (NA = 0.16), 10× (NA = 0.45), 20× (NA = 0.8) and 40× (NA = 0.95) (Carl Zeiss Microscopy), motorized specimen stage (MBF Bioscience, Williston, VT, USA), stage controller (MAC 6000 XY; Ludl Electronics), focus encoder (MT 1271; Heidenhain, Traunreut, Germany), CCD color video camera (1600 × 1200 pixels; MBF Bioscience), and stereology software (Stereo Investigator Version 11.01.2 64 bit; MBF Bioscience).

**Figure 23:**
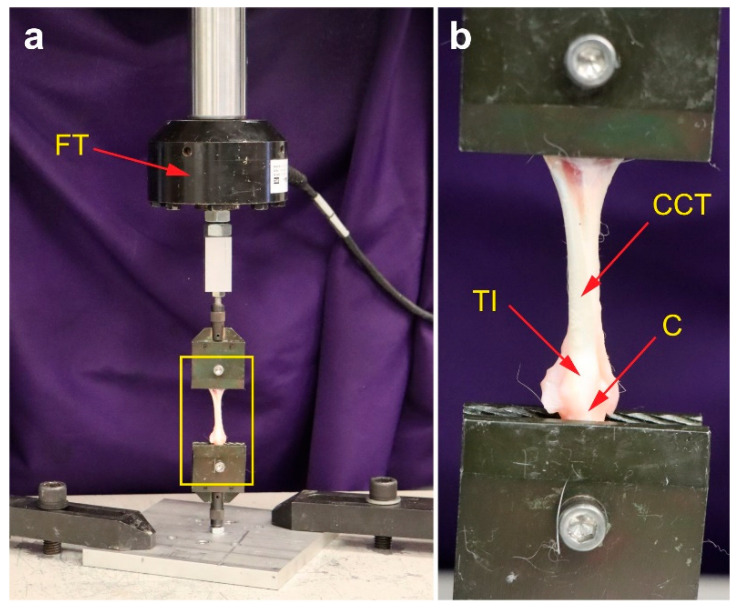
Non-destructive biomechanical analysis of a rabbit common calcaneal tendon ex vivo using a servo-hydraulic material testing system (MiniBionix 858; MTS Systems, Eden Prairie, MN, USA). The yellow rectangle in (**a**) indicates the position of the detail shown in (**b**). Abbreviations: FT, force transducer; CCT, common calcaneal tendon; TI, tendon insertion; C, calcaneus.

Biomechanical testing was conducted on limbs from Groups 5 and 6. Tendons with muscle remnants and attached calcaneus were dissected, and the tendon length and cross-sectional area (assuming rectangular geometry) were measured. Samples were kept hydrated during testing. Tendon constructs were mounted on a servo-hydraulic testing system (MTS MiniBionix 858; MTS Systems, Eden Prairie, MN, USA), with the calcaneus fixed and the tendon gripped at the proximal end (Figure 23).

The testing protocol included a 2 N pre-load, followed by 10 preconditioning cycles (0–2% strain), and a 5% engineering strain stress-relaxation test held for 100 s. Force and displacement were recorded to calculate peak load, equilibrium load, peak stress, equilibrium stress and percent relaxation.

Statistical analysis involved Fisher’s exact test to compare histological findings, and two-way ANOVA (two treatments; two time points), followed by Bonferroni’s multiple comparison test (UA-ADRCs vs. RLS), for stereologic analysis across Groups 1–4. One-way ANOVA followed by Bonferroni’s multiple comparison test was used to compare biomechanical data between left (intact) and right (treated/sham-treated) limbs in Groups 5 and 6. Post hoc power analysis and sample size calculations were performed using the Python module statsmodels (Version 0.14.4; [117]), accessed via ChatGPT 4.0. Linear regression analysis tested associations between stereologic or biomechanical results and the number of injected cells. A *p*-value < 0.05 was considered statistically significant. Analyses were conducted using GraphPad Prism (Version 10.1.2; GraphPad Software, San Diego, CA, USA).

All photomicrographs were captured digitally. Most photomicrographs were created from virtual slides (using the software Biolucida Viewer; Version 2020.1.0; MBF Bioscience) that were produced by digital photography using an automated scanning microscopy workstation. The latter consisted of an M2 AxioImager microscope (Carl Zeiss Microscopy), 10× Plan-Apochromate objective (NA = 0.3; Carl Zeiss Microscopy), 2-axis computer-controlled stepping motor system (4”× 3” XY; Prior Scientific, Cambridge, UK), focus encoder (Heidenhain) and color digital camera (AxioCam MRc; 2/3” CCD sensor, 1388 × 1040 pixels; Carl Zeiss Microscopy). The whole system was controlled by the software Stereo Investigator (Version 11.06.2; MBF Bioscience). On average 670 (range, 211–953) images were captured for each composite. These images were made into one montage each using the Virtual Slide module of the Stereo Investigator software (MBF Bioscience); the size of the resulting 2D virtual slides varied between 67 MB and 359 MB.

Polarization photomicrographs were produced using an Axiophot Microscope (Carl Zeiss Microscopy) equipped with an Axiocam HRc digital camera (2/3” CCD sensor, 1388 × 1040 pixels; Carl Zeiss Microscopy) that was controlled by the software Zeiss Axiovision SE64 (Rel. 4.9.1 SP2; Carl Zeiss Microscopy). The images were taken in transmitted light mode either without or with polarized light using a 5× Plan-Neofluar objective (NA = 0.15; Carl Zeiss Microscopy). The polarized images were taken in black and white mode of the digital camera. Illumination was adjusted using the automatic measurement function of the Zeiss Axiovision software.

The final figures were constructed using Corel Photo-Paint 2021 and Corel Draw 2021 (both versions 23.1.0.389; Corel, Ottawa, ON, Canada). Only adjustments of contrast and brightness were made using Corel Photo-Paint, without altering the appearance of the original materials.

## 5. Conclusions

The present study shows, for the first time, that a single injection of UA-ADRCs into a partial-thickness Achilles tendon defect in rabbits leads to the formation of biomechanically functional, histologically organized new connective tissue with significantly better structural integration and less scar formation than after sham treatment.

This result has important implications for the development of minimally manipulated, autologous cell-based therapies that aim to restore tendon structure and function, potentially offering a truly regenerative and structure-modifying treatment option for partial tendon tears.

## Figures and Tables

**Table 1:** Results (*p*-values) of the statistical analysis of the data shown in Figure 19 with two-way ANOVA.

Two-Way ANOVA (Source of Variation)	Variable:	Cells	Vessels	ECM	Artifacts
Interaction (Time × Treatment)		0.021	0.019	0.112	0.705
Time		0.001	0.022	0.006	0.567
Treatment		0.228	0.001	0.385	0.324
95% confidence interval of mean difference at W4		−0.0755 to 0.170	−0.0195 to 0.0520	−0.136 to 0.0864	−0.138 to 0.0603
95% confidence interval of mean difference at W12		−0.256 to −0.0105	0.0343 to 0.106	−0.0309 to 0.192	−0.117 to 0.0816

Abbreviation: ECM, extracellular matrix.

**Table 2:** Effect size (Cohen’s f) and observed power (OP) from the one-way ANOVA of the data in Figure 20, along with the total number (n_Total_) and per-group number (n_Group_) of rabbits required to achieve 80% power for the same effect size across four groups.

	Peak Load	Equilibrium Load	Peak Stress	Equilibrium Stress	Percent Relaxation	Cross-Sectional Area
Effect size	0.29	0.27	0.4	0.3	0.54	0.45
OP [%]	21.8	19.4	39.4	23.2	65.5	49.3
n_Total_	135	155	73	126	42	58
n_Group_	34	39	18	32	11	15

**Table 3:** Details of studies on treatment of experimentally induced tendon injuries with autologous, cultured, adipose-derived stem cells in vivo.

R	Y	Sp	T	I	P	S	AT	C	H	PM	IHC	B
[55]	2011	Rabbit	CCT	Incision	+	-	PRP	-	+	-	-	-
[56]	2012	Rabbit	CCT	Transection	+	-	PRP	PRP alone	+	-	+	+
[57]	2013	Horse	SDFT	Collagenase gel	-	-	PC	Sham	+	-	-	-
[58]	2014	Horse	SDFT	Lesion created by a standardized surgical model	-	-	-	Sham	+	-	+	-
[59]	2014	Rabbit	CCT	Resection of a 2 cm-long tendon fragment	-	+	-	Cell-free scaffold	+	--	-	+
[7]	2014	Rabbit	SST	Sharp release of the insertion of the SST at the greater tuberosity	+ ^1^	-	-	Sham	-	-	+	-
[60]	2016	Horse	SDF	Lesion created by a standardized surgical model	-	-	-	Sham	+	-	+	-
[61]	2016	Dog	FDPT	Transection	+	+	GF	Cell-free scaffold	-	-	-	+
[62]	2016	Dog	FT	Transection	+	+	-	Cell-free scaffold	-	-	+	-
[63]	2017	Horse	SDFT	Lesion created by a standardized surgical model	-	-	PRP	BM-MSCs	+	-	-	-
[64]	2017	Horse	SDFT	Lesion created by a standardized surgical model	-	-	-	Sham	+	-	-	+
[65]	2017	Dog	FDPT	Transection	+	+	GF	Cell-free scaffold	+	-	+	-
[66]	2018	Horse	SDFT	Collagenase	-	-	-	Autologous serum	+	+	+	-
[67]	2019	Rabbit	PT	Partial patellectomy	+	+	US	No cells, no US	+	+ ^3^	-	+
[68]	2019	Rabbit	FDST	Transection	+	-	-	Sham	-	-	-	+
[69]	2019	Sheep	AT	Collagenase	-	-	rESWT	PRP	+	-	+	-
[70]	2020	Rat	SST	Sharp release of the insertion of the SST at the greater tuberosity	+ ^2^	+	-	Cell-free scaffold	+	-	+	+

Abbreviations: R, reference number; Y, year of publication; Sp, species; T, tendon; I, injury; P, primary repair (+, yes; -, no); S, use of a scaffold (+, yes; -, no); AT, additional treatment; C, control treatment; H, histology (+, yes; -, no); PM, polarization microscopy (+, yes; -, no); IHC, immunohistochemistry (+, yes; -, no); B, biomechanical analysis (+, yes; -, no); CCT, common calcaneal tendon; SDFT, superficial digital flexor tendon; SST, supraspinatus tendon; FDPT, flexor digitorum profundus tendon; FT, flexor tendon; PT, patella tendon; FDST, flexor digitorum superficialis tendon; AT, additional treatment (-, no additional treatment); PRP, platelet rich plasma; PC, platelet concentrate; GF, growth factors; US, low-intensity pulsed ultrasound; rESWT; radial extracorporeal shock wave therapy. ^1^ three weeks after release of the insertion of the supraspinatus tendon at the greater tuberosity. ^2^ two weeks after release of the insertion of the supraspinatus tendon at the greater tuberosity. ^3^ polarization microscopy of the tendon–bone junction.

**Table 4:** Comparison of cell viability and regenerative cell proportions in human lipoaspirate processed using various commercially available enzymatic isolation systems.

	Cell Type	MSCs [%]	EP [%]	M2 [%]	CV [%]
	Surface Markers	CD45- CD31- CD34+	CD45- CD31+ CD34+	CD45+ CD206+	
Reference	System/Method				
[5]	A	32.8	15.3	16.4	85.2
[4]	A	20.0	--	--	--
[99]	B	16.1	9.4	5.6	82.0
[100]	C	10.7	--	--	84.0
[100]	D	9.1	--	--	82.0
[100]	B	8.9	--	--	69.3
[98]	E	7.2	--	--	50.3

Abbreviations: MSCs, mesenchymal stem/stromal cells; EP, endothelial progenitors; M2, M2-macrophages; CV, cell viability. System/method: A, Transpose RT system (InGeneron); B, GID SVF-2 system (GID Bio, Inc., Louisville, CO, USA); C, Cytori StemSource 900/MB system (Lorem Cytori USA, Inc., San Diego, CA, USA); D, PNC MultiStation (PNC Technologies Co., Ltd., Anyang, Republic of Korea); E, MediKhan Lipokit Platform (Medi Khan Inc., Seoul, Republic of Korea).

**Table 5:** Groups of rabbits investigated in the present study.

Group	n	Treatment	Type	Time Post-Treatment
1	4	UA-ADRCs	H/I	W4
2	4	UA-ADRCs	H/I	W12
3	4	RLS	H/I	W4
4	4	RLS	H/I	W12
5	8	UA-ADRCs	B/I	W12
6	8	RLS	B/I	W12

Abbreviations: n, number of animals; UA-ADRCs, uncultured, unmodified, autologous, adipose-derived regenerative cells; RLS, Ringer’s lactate solution; H, histology; I, immunohistochemistry; B, non-destructive biomechanical analysis; W4, four weeks post-treatment; W12, twelve weeks post-treatment.

**Table 6:** Characteristics of the antibodies used in this study.

Antibody	Characteristic	Specification
Procollagen 1		
Immunoglobuline isotype/clone status		IgG1/mouse, monoclonal
Catalog no./provider		SP1.D8/DSHB ^a^
Demasking of antigen		Not applicable
Blocking		Vector Bloxall SP-6000 ^b^, LOT ZG1216 from 4 April 2021, and normal horse serum blocking solution 2.5% (S-2012-50) ^b^
Dilution and incubation parameters		1:10, 4 °C, over night
Secondary antibody used		Horse-anti-mouse IgG BA-2000 ^b^, 1:200
Type III collagen		
Immunoglobuline isotype/clone status		IgG1/mouse, monoclonal
Catalog no./provider		C7805 (Clone FH-7A)/Sigma-Aldrich (St. Louis, MO, USA)
Demasking of antigen		Protease XIV
Blocking		Vector Bloxall SP-6000 ^b^, LOT ZG1216 from 4 April 2021, and normal horse serum blocking solution 2.5% (S-2012-50) ^b^
Dilution and incubation parameters		1:150, 4 °C, over night
Secondary antibody used		Horse-anti-mouse IgG BA-2000 ^b^, 1:200
CD163		
Immunoglobuline isotype/clone status		IgG1/mouse, monoclonal
Catalog no./provider		5C6 FAT BMA Biomedicals (Augst, Switzerland)
Demasking of antigen		Not applicable
Blocking		3% H_2_O_2_ in Methanol
Dilution and incubation parameters		1:400, 4 °C, over night
Secondary antibody used		Horse-anti-mouse IgG BA-2000 ^b^, 1:200
Aggrecan		
Immunoglobuline isotype/clone status		IgG1/mouse, monoclonal
Catalog no./provider		12/21/1-C-6/DSHB ^a^
Demasking of antigen		3% H_2_O_2_ in Methanol/Chondroitinase AC ^c^
Blocking		Normal horse serum blocking solution 2.5% (S-2012-50) ^b^
Dilution and incubation parameters		1:5, 4 °C, over night
Secondary antibody used		Horse-anti-mouse IgG BA 2000 ^b^, 1:200

^a^ Antibodies SP1.D8 (developed by Dr. H. Furthmayr) and 12/21/1-C-6 (developed by Dr. B. Caterson) were obtained from the Developmental Studies Hybridoma Bank (DSHB), created by the Eunice Kennedy Shriver National Institute of Child Health and Human Development of the National Institutes of Health of the United States, and maintained at The University of Iowa, Department of Biology, Iowa City, IA, USA. ^b^ Provider: Vector Laboratories (Burlingame, CA, USA). ^c^ The 12/21/1-C-6 antibody recognizes an epitope on the core protein of the aggrecan molecule. Therefore, the pre-treatment requires the removal of the disaccharide side chains and the removal of oxidation effects [42].

## Data Availability

All raw data are available from the corresponding author on reasonable request.

## Abbreviations

The following abbreviations are used in this manuscript:

sPTRCT	Symptomatic, partial-thickness rotator cuff tears
UA-ADRCs	Uncultured, unmodified, autologous, adipose-derived regenerative cells
RCT	Randomized clinical trial
CCT	Common calcaneal tendon
RLS	Ringer’s lactated solution
ECM	Extracellular matrix
PBS	Phosphate-buffered saline
